# Peptides derived from MARCKS block coagulation complex assembly on phosphatidylserine

**DOI:** 10.1038/s41598-017-04494-y

**Published:** 2017-06-27

**Authors:** Noah Kastelowitz, Ryo Tamura, Abimbola Onasoga, Timothy J. Stalker, Ormacinda R. White, Peter N. Brown, Gary L. Brodsky, Lawrence F. Brass, Brian R. Branchford, Jorge Di Paola, Hang Yin

**Affiliations:** 1 Department of Chemistry & Biochemistry and the BioFrontiers Institute, University of Colorado Boulder, Boulder, Colorado USA; 2 Department of Pediatrics, University of Colorado School of Medicine, Aurora, Colorado USA; 3 Department of Medicine, University of Pennsylvania, Philadelphia, Pennsylvania USA

## Abstract

Blood coagulation involves activation of platelets and coagulation factors. At the interface of these two processes resides the lipid phosphatidylserine. Activated platelets expose phosphatidylserine on their outer membrane leaflet and activated clotting factors assemble into enzymatically active complexes on the exposed lipid, ultimately leading to the formation of fibrin. Here, we describe how small peptide and peptidomimetic probes derived from the lipid binding domain of the protein myristoylated alanine-rich C-kinase substrate (MARCKS) bind to phosphatidylserine exposed on activated platelets and thereby inhibit fibrin formation. The MARCKS peptides antagonize the binding of factor Xa to phosphatidylserine and inhibit the enzymatic activity of prothrombinase. In whole blood under flow, the MARCKS peptides colocalize with, and inhibit fibrin cross-linking, of adherent platelets. *In vivo*, we find that the MARCKS peptides circulate to remote injuries and bind to activated platelets in the inner core of developing thrombi.

## Introduction

Phosphatidylserine (PS) is an essential cofactor of the coagulation proteins involved in hemostasis and thrombosis. PS is an anionic lipid that is normally sequestered to the inner leaflet of the plasma membranes and only exposed on the outer membrane surface during activated processes like apoptosis or platelet activation. During blood clotting, activated platelets undergo changes in membrane shape and expose PS on their outer membrane leaflet via calcium-dependent phospholipid scramblases^1–3^. Activated coagulation factors in the presence of calcium and PS assemble into enzymatically active complexes, e.g., prothrombinase, a complex of activated factor Xa (FXa) and Va (FVa)^4–10^. The enzymatic activity of these coagulation protein complexes ultimately leads to the formation of fibrin, an insoluble polymer that forms an affixing crosslinking mesh around the hemostatic platelet plug.

In this work, we explore the effect of small PS-binding peptides on blood coagulation. The peptides are based on the protein myristoylated alanine-rich C kinase substrate (MARCKS), a 32 kDa protein that sits at the interface of many lipid-protein interactions. Full length MARCKS protein has two important known functional domains: a myristoylated N-terminus that inserts into, and localizes the protein to, the inner leaflet of the plasma membrane, and a highly basic effector domain that mediates its biologic functions by reversibly binding the plasma membrane^11^. Phosphorylation of the effector domain by protein kinase C, or binding by calmodulin in the presence of calcium, inhibit the domain’s association to the plasma membrane, releasing sequestered phosphoinositides and disrupting the actin network around the membrane^12–16^. MARCKS is expressed across most tissues in the body, including platelets, and the actions of the MARCKS have been implicated in neurodevelopment, cell motility, and endo- and exocytosis^17–21^.

**Figure 1:**
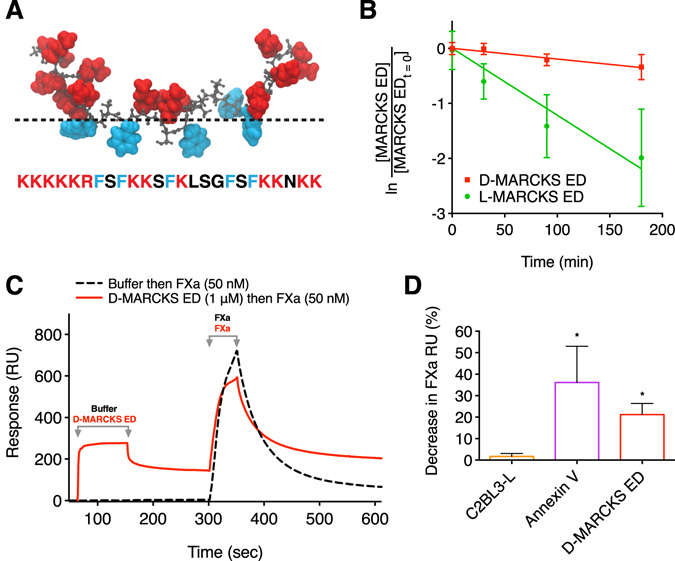
D-MARCKS ED is a protease resistant peptide that antagonizes the binding of FXa to PS. (**A**) Sequence and representative membrane bound conformation of the MARCKS ED peptide. Positively charged residues are shown in red and phenylalanine residues are shown in blue. Dashed line shows approximate location of the membrane lipid head groups. (**B**) Comparison of the human serum stability of L-MARCKS ED (*t*_1/2_ = 57 min, 95% confidence interval (CI) 46 to 79 min) and D-MARCKS ED (*t*_1/2_ = 356 min, 95% CI 252 to 606 min) peptides (*n* = 3, mean ± SD). (**C**) Representative reference corrected Biacore 3000 SPR sensogram showing D-MARCKS ED antagonizes the binding of FXa to lipid membrane surface containing PS. Dashed black line shows an injection of running buffer at t = 30 sec and an injection of 50 nM FXa at t = 300 sec. Following complete dissociation of FXa from the membrane surface, the solid red line shows an injection of 1 μM D-MARCKS ED at t = 30 sec and an injection of 50 nM FXa at t = 300 sec. (**D**) Quantification of FXa SPR binding inhibition with BiOptix 404pi instrument by negative control C2BL3-L, positive control annexin V, and D-MARCKS ED (*n* = 3 C2BL3-L and annexin V; *n* = 5 D-MARCKS ED, mean ± SD). *P < 0.05 compared to C2BL3-L by Kruskal-Wallis test followed by Dunn’s *post hoc* test.

We have previously demonstrated that peptides based on the effector domain of MARCKS (MARCKS ED) bind with increasing affinity to lipid membranes that are both highly curved and enriched in PS^22^. MARCKS ED does not adopt a secondary structure. Rather, the peptide forms a random coil conformation that selectively binds through electrostatic attraction between its 13 basic residues and the acidic phosphatidylserine lipid head groups, and the insertion of bulky phenylalanine residues into the hydrophobic membrane core (Fig. 1A)^23–25^. The affinity of MARCKS ED for curved and PS enriched membranes is sequence specific, but independent of the amino acid stereo conformation^24, 25^. MARCKS ED has a poor affinity for phosphatidylcholine (PC) membranes and its affinity for PS enriched membranes is increased by the presence of phosphatidylethanolamine (PE)^22, 24^. MARCKS ED is less than a tenth of the molecular weight of PS binding proteins annexin V and lactadherin, and comparatively easy to synthesize and modify using solid phase peptide synthesis. Moreover, unlike annexin V, MARCKS ED does not require calcium to bind to PS^22^.

Having established the affinity of MARCKS ED for PS, we sought here to determine if the peptide could affect the binding of a coagulation factor to PS. We hypothesized that MARCKS ED would bind to the curved membranes and exposed PS of activated platelets. We establish that the MARCKS ED peptide can antagonize the interaction between FXa and PS, and that this activity significantly reduces FXa enzymatic activity. We find that MARCKS ED only binds to platelets that are activated and, in whole blood, inhibits the formation of fibrin, the end product of the coagulation cascade. Finally, we show *in vivo* that MARCKS ED can circulate to a remote injury site and bind to activated platelets in the core of a developing thrombus. We establish that a peptide can antagonize a lipid-protein interaction, and that this activity can have significant effects in a complex multicomponent pathway like blood coagulation.

## Results

### D-MARCKS ED is protease resistant

Peptides in blood are exposed to an array of proteases that may reduce their biologic activity^26^. Since MARCKS ED binding is independent of amino acid chirality, the peptidomimetic D-amino acid form of MARCKS ED has the potential comparative advantage of increased resistance to protease degradation and reduced likelihood of native biologic recognition or activity^24, 25^. We first sought to confirm that D-MARCKS ED is the more stable isoform to proteolysis. Separate incubation of the two peptides with human plasma resulted in pseudo-first-order degradation of the peptides (Fig. 1B). We observed the half-life of D-MARCKS ED to be roughly six times longer than that of L-MARCKS ED. Based on this result, we chose to use D-MARCKS ED for studies where only a single isoform could be reasonably tested.

### D-MARCKS ED antagonizes binding of FXa to PS

To investigate if MARCKS ED could antagonize the binding of a coagulation factor to PS, we used surface plasmon resonance (SPR). Buffer or D-MARCKS ED was flowed over an immobilized lipid surface containing PS. After 160 seconds, FXa was then injected over the lipids and the binding response was recorded. Pretreatment of the same surface with D-MARCKS ED resulted in a 50% ( ± SD of 5%) mean reduction in FXa membrane binding as compared to the buffer treatment using a Biacore 3000 instrument (Fig. 1C and Supplementary information, Fig. S1A). We repeated this experiment on a BiOptix 404pi instrument. Compared to the Biacore 3000 instrument, the BiOptix 404pi instrument absorbed approximately six times more lipid reference units (RU) to the SPR HPP chip surface (not shown) and showed reduced FXa binding inhibition by D-MARCKS ED (Fig. 1D and Supplementary information, Fig. S1B) with equivalent injection concentrations, association volumes, and flow rates. To validate these results, annexin V was also tested to serve as a positive control and C2BL3-L (NH_2_-GGDYDKIGKNDA-CONH_2_), a small peptide previously shown to have poor affinity for PS, was tested to serve as a negative control (Fig. 1D and Supplementary information, Fig. S1C and D)^27^.

The observed difference in FXa binding inhibition by D-MARCKS ED with the two instruments likely relates to the stoichiometry of the MARCKS ED/PS interaction and the resulting relative PS surface coverage of D-MARCKS ED in each experiment. Previous work investigating the interaction of D-MARCKS ED with the lipid phosphatidylinositol 4,5-bisphosphate (PIP_2_) has shown that the stoichiometry of the interaction is determined by electrostatic complementarity between the peptide and lipids^28, 29^. The D-MARCKS ED peptide has a net positive charge of 13 at physiologic pH, suggesting each D-MARCKS ED peptide could bind and block up to 13 PS lipids. Using this stoichiometry and the RU definition of both SPR instruments (1000 RU = 1 ng mm^−2^), we make the approximation that D-MARCKS ED blocked 82% ( ± SD of 9%) of PS in the Biacore 3000 experiment and this resulted in a 50% ( ± SD of 5%) reduction in FXa binding. In comparison, with the increased lipid loading seen with the BiOptix 404pi, we approximate that D-MARCKS ED blocked 7% ( ± SD of 3%) of PS and this resulted in a 23% ( ± SD of 5%) reduction in FXa binding in the BiOptix 404pi experiment. Since the injections of D-MARCKS ED and FXa are separated temporally, to resolve the binding response of each ligand, and spatially, by the continuous flow of buffer over the chip, both ligands cannot simultaneously saturate the membrane surface. We therefore cannot infer stoichiometry of the inhibition, but do observe that more D-MARCKS ED surface coverage results in reduced FXa binding, supporting our initial hypothesis that MARCKS ED could antagonize the binding of a coagulation factor to PS.

### MARCKS ED binds to activated platelets and inhibits prothrombinase

**Figure 2:**
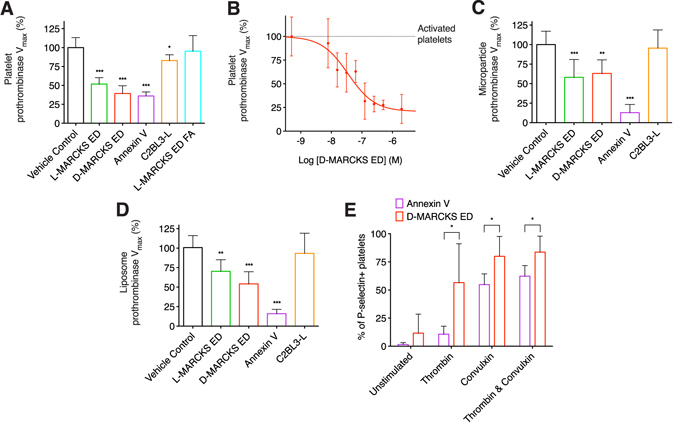
MARCKS ED inhibits prothrombinase enzymatic activity in the presence of PS containing membranes and only binds to activated platelets. (**A**) Prothrombinase activity of washed human platelets stimulated with thrombin and convulxin when pre-treated with vehicle control or 1 μM L-MARCKS ED, D-MARCKS ED, positive control annexin V, negative control C2BL3-L, or negative control L-MARCKS ED FA mutant (*n* = 8, mean ± SD). (**B**) Dose response of D-MARCKS ED pre-treatment on the prothrombinase activity of washed human platelets stimulated with convulxin and thrombin (*n* = 4, mean ± SD). (**C**) Prothrombinase activity of exosomes isolated from MDA-MB-231 cells when pre-treated with vehicle control or 1 μM L-MARCKS ED, D-MARCKS ED, annexin V, or C2BL3-L (*n* = 8, mean ± SD). (**D**) Prothrombinase activity of synthetic liposomes composed of POPC/POPS at a 19/1 ratio when pre-treated with vehicle control or 1 μM L-MARCKS ED, D-MARCKS ED, annexin V, or C2BL3-L (*n* = 8, mean ± SD). (**E**) The binding of annexin V (Brilliant Violet 605) and D-MARCKS ED (NBD) to P-selectin (Cy5) positive platelets was compared by flow cytometry when the platelets were left unstimulated or stimulated with thrombin, convulxin, or thrombin and convulxin (*n* = 6, mean ± SD). *P < 0.05, **P < 0.01, ***P < 0.001 either by comparison to vehicle control by ANOVA followed by Dunnet’s *post hoc* test (**A**,**C**,**D**) or two tailed Student’s *t*-test (**E**).

To determine if antagonizing the binding of FXa to PS with MARCKS ED resulted in reduced enzymatic activity, we used a platelet-dependent prothrombinase assay. In the presence of human platelets activated by convulxin and thrombin, L- and D-MARCKS ED both significantly reduced prothrombinase activity (Fig. 2A). Given the 17% reduction in prothrombinase activity observed with our negative control peptide C2BL3-L, L-MARCKS ED FA (a MARCKS ED peptide where membrane affinity is reduced by replacing all phenylalanine residues with alanine) was added as an additional negative control and did not show any inhibition (Fig. 2A)^22^. Interestingly, a dose response curve with D-MARCKS ED showed the peptide could not completely inhibit the formation of thrombin (Fig. 2B). It has been shown previously that activation of platelets by convulxin and thrombin generates a unique subpopulation of approximately 30% of activated platelets that express high levels of procoagulant proteins – including membrane surface bound FVa, likely originating in the platelet α granules^30, 31^. As the binding of this membrane bound FVa cannot be antagonized, D-MARCKS ED may be unable to completely block prothrombinase assembly and activity resulting from this subpopulation.

We also examined the effect of MARCKS ED on prothrombinase activity in the presence of other PS-containing membranes. We repeated the prothrombinase assay, omitting the addition of platelet agonists convulxin and thrombin, using microparticles isolated from MDA-MB-231 human breast cancer cells as well as with similarly sized extruded synthetic liposomes containing PS (Supplementary information, Fig. S2A and B). Microparticles were selected due to the proposed importance of PS exposure on tumor microparticles during cancer related venous thromboembolism^32^. L- and D-MARCKS ED both inhibited microparticle dependent prothrombinase activity (Fig. 2C). The membranes of platelets and microparticles both contain transmembrane proteins, most noteworthy for this study, tissue factor. Therefore, synthetic liposomes were used to provide an assembly surface free of any membrane proteins. The observed relative prothrombinase inhibition using the synthetic liposomes (POPC/POPS at a 19/1 molar ratio) was similar to that seen with the microparticles (Fig. 2D). This result, along with the inhibition of FXa binding demonstrated by SPR, strongly suggests that MARCKS ED inhibits prothrombinase activity by directly blocking the binding of FXa to PS.

### D-MARCKS ED only binds to activated platelets

To determine if MARCKS ED bound only to activated platelets, we analyzed D-MARCKS ED and annexin V binding to platelets in the presence of a variety of activating agonists using flow cytometry. Neither D-MARCKS ED nor annexin V labeled the unstimulated platelets (Fig. 2E and Supplementary information, Figs S3 and S4). When platelets were activated with thrombin, convulxin, or thrombin and convulxin, D-MARCKS ED labeled a greater percentage of the P-selectin positive platelets than annexin V (Fig. 2E). The combination of thrombin and convulxin activation resulted in the greatest percentage of P-selectin positive platelets labeled with D-MARCKS ED or annexin V (Fig. 2E). We observed a strong correlation between relative P-selectin positivity and D-MARCKS ED binding (Supplementary information, Fig. S3). D-MARCKS ED fluorescence intensity was normally distributed and continuously increased with greater platelet activation (Supplementary information, Fig. S4A). In contrast, annexin V fluorescence intensity showed a bimodal distribution that shifted into the more fluorescent population with greater platelet activation (Fig. S4B). This difference could be due to the membrane PS composition threshold required for annexin V binding, previously described as 2.5 to 8%, that is not required for MARCKS ED binding^24, 33^.

### MARCKS ED inhibits fibrin formation in whole blood

**Figure 3:**
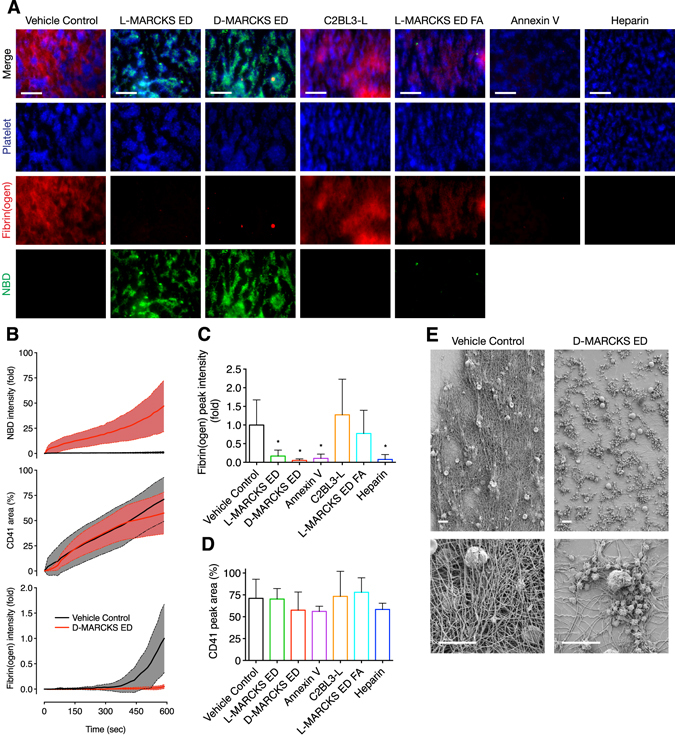
MARCKS ED inhibits fibrin formation in whole blood under physiologic flow conditions. (**A**) Representative images of platelet accumulation (blue, anti-CD41), fibrin(ogen) formation (red, Alexa Flour 647-fibrinogen), and peptide binding (green, NBD-peptide) in the whole blood microfluidic flow assay after 10 min at wall shear rate 100 s^−1^ when pre-treated with vehicle control or 1 μM L-MARCKS ED, D-MARCKS ED, negative control C2BL3-L, negative control L-MARCKS ED FA mutant, positive control annexin V, or positive control 15 USP ml^−1^ heparin (*n* = 6). Scale bar, 50 μm. (**B**) Time course of peptide NBD fluorescence intensity, platelet surface area coverage, and fibrin(ogen) intensity for the vehicle control and D-MARCKS ED treatment in the microfluidic flow assay (*n* = 6, mean ± SD). (**C**) Final fibrin(ogen) intensity values for each treatment in the microfluidic flow assay (*n* = 6, mean ± SD). (**D**) Final platelet surface coverage values for each treatment in the microfluidic flow assay (*n* = 6, mean ± SD). (**E**) Representative scanning electron micrographs of finals clots formed in microfluidic flow assay with vehicle control and D-MARCKS ED treatment (*n* = 3). Scale bar, 10 μm. *P < 0.05 compared to vehicle control by ANOVA followed by Dunnet’s *post hoc* test (**C**,**D**).

Next, we assessed the effects of the MARCKS ED peptides on fibrin formation in whole blood. Using a custom microfluidic device, whole human blood was flowed over an immobilized fibrilar collagen strip at a venous wall shear rate^34^. Treatment with a vehicle control resulted in the formation of a dense fibrin mesh on the adherent platelets (Fig. 3A and Supplementary information, Video S1). When the blood was pre-incubated with L- or D-MARCKS ED peptide, no fibrin mesh formation was observed (Fig. 3A–C, and Supplementary information, Fig. S5A, and Video S2). Treatment with either negative control peptide did not inhibit the formation of fibrin, while positive controls annexin V and heparin showed equivalent fibrin formation inhibition to that of the MARCKS ED peptides (Fig. 3A,C and Supplementary information, Fig. S5B–E). No treatment appreciably changed the surface coverage percentage of the platelets, suggesting the peptides did not interfere with the interaction between platelets, von Willebrand factor, or collagen (Fig. 3D). Of the NBD labeled peptides, only L- and D-MARCKS ED showed significant fluorescence intensity and clear colocalization with the adherent platelets (Fig. 3A and Supplementary information, Fig. S6). To confirm the fibrin formation inhibition visualized by fluorescence, the vehicle control and D-MARCKS ED final clots were fixed and visualized by scanning electron microscopy. Clots formed with vehicle control treatment showed the formation of dense fibrin mesh over the adherent platelets, while D-MARCKS ED treated samples exhibited exposed fibrillar collagen and platelet clumps, and no visible fibrin formation (Fig. 3E).

### D-MARCKS ED binds to thrombi *in vivo*

**Figure 4:**
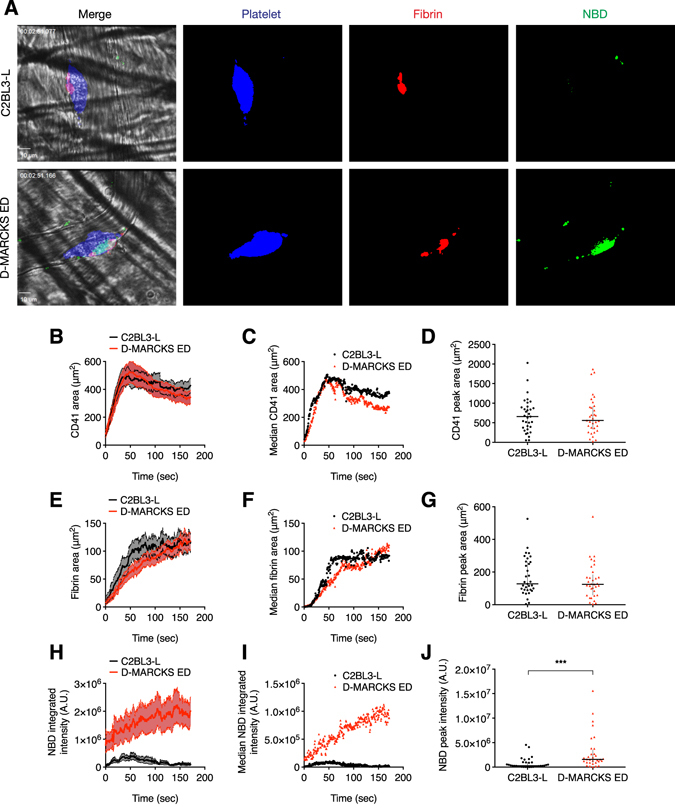
D-MARCKS ED binds to thrombi *in vivo*. (**A**) Representative fluorescence and bright field images of platelet accumulation (blue, anti-CD41), fibrin formation (red, anti-fibrin), and peptide binding (green, NBD-peptide) in the murine intravital laser-induced microvascular injury model when treated with 5 mg kg^−1^ negative control C2BL3-L or D-MARCKS ED. Scale bar, 10 μm. (**B**–**J**) Platelet area, fibrin area, and peptide NBD integrated fluorescence intensity accumulation following murine intravital microvascular injury when treated with 5 mg kg^−1^ C2BL3-L or D-MARCKS ED. Time course data is shown as mean ± SEM (**B**,**E**,**H**) and median (**C**,**F**,**I**) values. Peak platelet, fibrin, and NBD values (**D**,**G**,**J**) are shown as mean ± SEM (*n* = 33 thrombi from 4 mice). ***P < 0.001 when compared by the Mann-Whitney statistical test (**D**,**G**,**J**).

To assess the *in vivo* localization of D-MARCKS ED, as well as its effects on the hemostatic response, we used a murine intravital laser-induced microvascular injury model^35^. Male mice were treated with 5 mg kg^−1^ C2BL3-L or D-MARCKS ED through a jugular vein cannula. 30 min later, fibrin and platelet deposition, as well as peptide fluorescence, were observed at a cremaster microcirculation injury. At the injury site, D-MARCKS ED localized primarily to the innermost area of the thrombus (Fig. 4A,H–J). This area is the core of thrombus, and the primary location of thrombin activity and fibrin formation^35^. Little or no C2BL3-L fluorescence was observed in the thrombi (Fig. 4A,H–J). The initial velocity of fibrin formation with D-MARCKS ED treatment appeared to be reduced, nonetheless, the difference in fibrin formation lag time, or peak platelet or fibrin area values, was not statistically significant (Fig. 4B–G, and Supplementary information, Fig. S7).

## Discussion

In this work we have explored how small lipid-binding peptide and peptidomimetic probes can bind to activated, PS-exposing platelets and affect the blood coagulation pathway. We found that D-MARCKS ED could antagonize binding of FXa to PS, and that this activity inhibited the enzymatic activity of coagulation factor complex prothrombinase in the presence of a variety of PS-containing membranes. In whole blood, we observed dramatic inhibition of fibrin formation during contact activation pathway-driven coagulation in a microfluidic flow assay. *In vivo*, we observed D-MARCKS ED binding to platelets in the thrombus core, but we did not see a significant effect on fibrin formation. Coagulation initiation in the laser-induced microvascular injury model is more complex and believed to be driven by the exposure of tissue factor at the injury site^36^. While it is possible that therapeutic concentrations of D-MARCKS ED did not reach the *in vivo* injury site, the initiation of the coagulation cascade through tissue factor and factor VIIa could also explain the differences in fibrin formation inhibition between the *ex vivo* and *in vivo* models. For example, inhibition of the PS-dependent assembly of coagulation protein complex intrinsic tenase (factor VIIIa and factor IXa) by D-MARCKS ED would further slow fibrin generation in models dependent on the contact activation pathway, but have lesser effect on coagulation initiated through the tissue factor pathway. The PS binding protein lactadherin has been previously shown to inhibit the procoagulant activity of PS *in vivo*, demonstrating this mechanism is a viable pathway to block hemostasis and thrombosis^37^.

Interwoven with coagulation cascade initiation is the concurrent activation of platelets through interaction with collagen. This creates a positive feedback loop where activated platelet PS exposure serves as a cofactor for thrombin generation, and the generated thrombin further activates platelets. The combination of collagen or convulxin, both of which bind the platelet GPVI receptor, with thrombin is the strongest activator of platelets and the most effective driver of platelet-dependent thrombin generation^38^. Strong activation causes the platelet membrane to balloon and bleb and exposes a high amount of PS, while weaker activation, e.g., only thrombin, leaves the platelet membrane comparatively intact and causes less PS exposure^9, 38, 39^. Unsurprisingly, D-MARCKS ED demonstrated the greatest degree of binding to those platelets that were activated by the combination of convulxin and thrombin. Similar activation conditions occur when platelets encounter the immobilized collagen in the microfluidic flow assay, and this resulted in D-MARCKS ED labeling of almost all adherent platelets and the inhibition of fibrin formation. Conversely, in the laser-induced microvascular injury model, only a small portion of the platelets in the thrombus core are bound by D-MARCKS ED. These are platelets directly proximal to the injury site and the most likely to be exposed to subendothelial collagen. These results suggest that the more robust platelet activation response that occurs from GPVI activation is the most favorable for D-MARCKS ED binding and potential fibrin formation inhibition.

Peptides derived from the full length MARCKS protein sequence have previously been shown to have a number of biomodulary effects. The MANS peptide, made up of the myristoylated N-terminal sequence of MARCKS, interferes with mucus hypersecretion, cancer metastasis, and proinflammatory cytokine expression^40–43^. MARCKS ED has been shown to reduce platelet serotonin release, block monocyte tissue factor initiated coagulation, and also interfere with proinflammatory cytokine expression^18, 43–45^. These activities have been suggested to occur through various mechanisms, including altering cytoskeleton dynamics and blocking phosphorylation of endogenous MARCKS protein^41, 42, 44^. We have shown here that the MARCKS ED peptide binds to physiologically exposed PS and antagonizes coagulation factor binding and enzymatic activity. We did not observe MARCKS ED labeling or internalization by unstimulated platelets, suggesting the peptide does not quickly penetrate the plasma membrane. It is possible that some of the previously described biologic effects of MARCKS-derived peptides are also due to binding of peptides to the outer membrane surface. As endogenous full length MARCKS is expressed in platelets, MARCKS associated to the inner leaflet of platelet plasma membranes may also be localized to the outer membrane surface during platelet activation, and could potentially have a physiologic role of modulating the platelet procoagulant response. Future investigation will be needed to explore this hypothesis.

Modulation of lipid protein interactions remains a challenging task and an essentially unexplored drug target^46^. In addition to the lock and key-like interaction of lipid head groups with proteins, the lipid membrane also has colligative physical properties, such as lipid packing, bilayer thickness, or surface charge, that change the environment of the interaction. PS binding proteins, such as annexin V and lactadherin, have previously been shown to compete with coagulation factors for PS binding sites leading to inhibition of coagulation protein complex enzymatic activity^37, 47–49^. Compared to these proteins, the MARCKS ED peptide is significantly smaller in size, more readily chemically modified during solid phase peptide synthesis, and, in the D-amino acid form, more stable to proteolysis and likely less immunogenic^50^. Perhaps most unique, the interaction between MARCKS ED and PS is not based on secondary or tertiary structure, but a primary structure-based interaction that occurs through a unique combination of peptide charge and hydrophobicity^23–25^. Our work here shows that this comparatively simple interaction motif is sufficient to inhibit the interaction between a coagulation factor and PS, and also specific enough to localize to a remote intravascular injury *in vivo*.

## Methods

### Investigational peptides synthesis

Peptides were synthesized using standard solid phase Fmoc chemistry on a Liberty microwave-assisted peptide synthesizer (CEM, Matthews, NC). Fluorescently labeled peptides were produced by conjugating NBD (4-chloro-7-nitrobenzo-2-oxa-1,3-diazole) via an aminohexanoic acid linker to the peptide N-terminus. Crude peptide was purified by a 1200 Series reverse phase HPLC (Agilent Technologies, Santa Clara, CA) using a semi-prep C18 column and characterized using either a Voyager DE-STR MALDI-TOF mass spectrometer (Applied Biosystems, Grand Island, NY) or a Synapt G2 HDMS Q-TOF mass spectrometer (Waters, Milford, MA) fitted with an electrospray ionization source. HPLC peptide eluates were collected, lyophilized to dryness, and stored at −20 °C.

### Serum stability

The serum stability of L- and D-MARCKS ED was measured as in a previously described protocol^51^. Briefly, 100 μg ml^−1^ L- or D-MARCKS ED was incubated at 37 °C in RPMI 1640 medium (Fisher Scientific, Pittsburgh, PA) supplemented with 25% (v/v) pooled normal human serum (Innovative Research, Novi, MI). At specific time points, the reaction was subsampled and a trichloroacetic acid solution was added to a final concentration of 5% (w/v). Each subsample mixture was cooled for 15 min at 4 °C, then centrifuged at 16,000 g for 4 min to precipitate the serum proteins. The resulting supernatant was analyzed with a 1200 Series reverse phase HPLC (Agilent Technologies) using a semi-prep C18 column. Non-degraded peptide was quantified by setting the UV-visible HPLC detection to 480 nm and integrating the chromatogram peaks with retention times matching that of the whole peptide (39 to 40 min). Peaks at 480 nm are due to the absorbance NBD fluorophore label. Subsamples from serum mixtures without added MARCKS ED showed no peaks in this retention time range.

### Synthetic liposome preparation

Synthetic liposomes were prepared using a modified version of a previously described method^52^. Briefly, chloroform suspended 1-palmitoyl-2-oleoyl-sn-glycero-3-phosphocholine (POPC), 1-palmitoyl-2-oleoyl-sn-glycero-3-phosphoethanolamine (POPE), and 1-palmitoyl-2-oleoyl-sn-glycero-3-phospho-L-serine (POPS) (Avanti Polar Lipids, Alabaster, AL) were combined to form lipid mixtures of the described molar ratios. Lipid solutions were then dried to a thin film under a slow N_2_ flow and vacuum desiccated for 1 hour. Lipid films were resuspended in HBS buffer (10 mM HEPES, 0.15 M NaCl, pH 7.4) containing 2 mM CaCl_2_. Homogenously sized liposomes were formed by extruding the lipid solutions through polycarbonate track etched membranes (GE Healthcare, Pittsburgh, PA) with pore sizes of 30, 100, and 400 nm using a LiposoFast FL-50 extruder (Avestin, Ottawa, Canada). Extruded liposome sizes were characterized by nanoparticle tracking analyses using a NanoSight LM14 (Malvern Instruments, Malvern, United Kingdom).

### Surface plasmon resonance

The effect of D-MARCKS ED on FXa binding to phosphatidylserine was assessed by SPR with Biacore 3000 (GE Healthcare) and 404pi (BiOptix, Boulder, CO) instruments. All experiments were run with HPP (alkyl-SAM) sensor chips (XanTec, Duesseldorf, Germany) in HBS running buffer containing 2 mM CaCl_2_. 500 μM liposome solutions were prepared and extruded through 100 nm pore size polycarbonate membranes as described above. After thorough flushing of the SPR instrument flow system and needles with 40 mM octyl glucoside (Sigma-Aldrich, St. Louis, MO) and running buffer, 150 μl of liposome solution was injected at a flow rate of 5 μl min^−1^ to coat the HPP sensor chip surface. Membrane surface formation was verified by injecting 50 μl of 0.1 mg ml^−1^ BSA at a flow rate of 5 μl min^−1^, where accumulation of less than 100 response units (RU) of BSA indicated formation of a complete self-assembled lipid monolayer^53^. Liposomes composed of POPC/POPS/POPE at a 3/5/2 molar ratio were used to form the experimental flow cell. The control flow cell, used for reference correcting, was formed with liposomes composed of only POPC. A flow cell coated only with BSA was used to verify that the peptides and proteins did not associate to the adsorbed BSA.

All subsequent injections were done at a flow rate of 60 μl min^−1^. Each competition experiment followed the same injection series; a 90 μl injection of either running buffer or 1 μM D-MARCKS ED, C2BL3-L, or annexin V (BioVision, Milpitas, CA), a 160 sec wait, followed by a 50 μl injection of 50 nM FXa (Haematologic Technologies, Essex Junction, VT). Complete regeneration of the membrane surface after injection of FXa was achieved with 10 μl of 2.5 M NaCl. Regeneration of the membrane surface after injection of D-MARCKS ED was not possible without using more stringent regeneration conditions that also partially removed the lipid surface. We therefore completely stripped the surface with 40 mM octyl glucoside and formed the membrane surface anew after each injection series of investigational peptide or protein. The absolute FXa binding response varied with each new membrane surface. As a result, investigational peptide or protein FXa binding inhibition was only calculated as compared to the uninhibited FXa binding response over the same formed membrane surface. Sensograms were analyzed with Scrubber2 (BioLogic Software, Campbell, Australia) using either reference correction to the only POPC surface to show the complete injection sequence (Fig. 1C), or a double referencing procedure for analysis of FXa response (Fig. 1D and Supplementary information, Fig. S1). Double referencing corrects the sensogram relative to both the response of the control flow cell and to buffer blank injections over the experimental flow cell^54^. In the case of these experiments, this corrects for the change in surface response to the buffer when it is pretreated with an investigational peptide or protein. The maximum SPR response was determined by averaging over the last 5 sec of the FXa association in the double referencing corrected sensograms.

### Whole blood collection and platelet preparation

Human whole blood was collected from healthy volunteers by venipuncture into 3.2% sodium citrate. Washed platelets were prepared from whole blood collected by venipuncture into 3.8% sodium citrate and acid-citrate-dextrose (ACD). Platelet-rich plasma (PRP) was prepared by centrifugation of whole blood at 200 *g* for 20 min. Platelets were isolated from the PRP by centrifugation at 1000 *g* for 10 min with 0.1 μg ml^−1^ prostacyclin (PGI_2_) (Sigma-Aldrich). The resulting pellet was resuspended with modified Tyrode’s buffer (129 mM NaCl, 20 mM HEPES, 12 mM NaHCO_3_, 2.9 mM KCl, 1.0 mM MgCl_2_, 0.34 mM Na_2_HPO_4_, 5 mM glucose, pH 7.3), ACD, and 0.1 μg ml^−1^ PGI_2_. Platelets were then washed by re-pelleting at 1000 *g* for 10 min and re-suspending with modified Tyrode’s buffer.

### Cell culture and exosome isolation

Biologic exosomes were isolated from MDA-MB-231 human breast cancer cells. Cells were grown to approximately 80% confluency on a 10 cm tissue culture dish. Culture media was then replaced with unsupplemented Dulbecco’s modified Eagle’s medium (Life Technologies, Grand Island, NY) and incubated for 2 days in a 1% oxygen environment. After incubation, exosomes were isolated from the cell media using Exoquick-TC (System Biosciences, Mountain View, CA) following the manufacturer’s standard protocol. The concentration (2.62 ± 0.65 × 10^11^ particles ml^−1^) and mean size (187 ± 66 nm) of the isolated exosomes was determined by nanoparticle tracking analyses using a NanoSight LM14 (Malvern Instruments).

### Prothrombinase assay

Effects on coagulation factor enzymatic activity were determined using a modification of a previously described prothrombinase assay^38, 55^. Washed human platelets (8 × 10^5^ reaction^−1^) were incubated at 37 °C with either a buffer blank or 375 ng ml^−1^ convulxin (Centerchem, Norwalk, CT) and 5.0 nM thrombin (Haematologic Technologies) in modified Tyrode’s buffer containing 2.9 mM CaCl_2_ and 0.05% wt vol^−1^ fatty acid-free bovine serum albumin (BSA). After 7 min, 1 μM investigational peptide or protein, or a buffer blank, was added, followed 3 min later by bovine FXa (3 nM) and FVa (6 nM) (Haematologic Technologies). 1 min later, 4 μM bovine prothrombin (Enzyme Research Laboratories, South Bend, IN) was added. After 4 min, the reaction mixture was subsampled into a Tris EDTA stop buffer (0.05 M Tris-HCl, 0.12 M NaCl, 2 mM EDTA, pH 7.5). The V_max_ of the generated thrombin was determined chromogenically by adding 0.5 mM S-2238 substrate (DiaPharma, West Chester, OH) and measuring changes in absorbance at 405 nm over time using a Synergy 2 microplate reader (BioTek, Winooski, VT). Effects on the prothrombinase activity of liposomes (100 nM POPC/POPS at a 19/1 molar ratio) and MDA-MB-231 exosomes (7 × 10^7^ reaction^−1^) were examined with identical conditions omitting the addition of convulxin and thrombin. At these concentrations, uninhibited thrombin generation by the liposomes and exosomes approximately matched that of the activated platelets. The half maximal inhibitory concentration (IC_50_) was fit by plotting [peptide] as log values and fitting with a nonlinear least-squares best-fit analysis using the equation prothrombinase V_max_ = V_max_^min^ + (V_max_^max^ − V_max_^min^)/(1 + 10^[Peptide]−logIC50^).

### Flow cytometry

Washed platelets were obtained from the whole blood as described above. The platelets were resuspended in modified Tyrode’s buffer containing 2 mM CaCl_2_, counted, and diluted to 2 × 10^4^ platelets μL^−1^. Platelets were stimulated with either 1 IU ml^−1^ thrombin (Chrono-log, Havertown, PA), 250 ng ml^−1^ convulxin (Centerchem), 1 IU ml^−1^ thrombin and 250 ng ml^−1^ convulxin, or left unstimulated for 10 min at room temperature. Platelets were then incubated for 30 min with Brilliant Violet 605 anti-human CD62P antibody (BioLegend, San Diego, CA), Cy5 annexin V (BD Biosciences, San Jose, CA), and 1 μM NBD labeled D-MARCKS ED peptide. The samples were then immediately fixed in 1% paraformaldehyde, diluted, and analyzed on a MoFlo Astrios EQ flow cytometer (Beckman Coulter, Brea, CA) with appropriate color compensation.

### Whole blood microfluidic flow assay

Effects on whole blood platelet activation and accumulation, as well as fibrin formation, were examined using a modification of a previously described microfluidic flow assay^34^. A custom polydimethylsiloxane microfluidic flow device containing four channels, each with a height of 100 μm and width of 500 μm, was vacuum mounted to a glass slide patterned with a type I fibrillar collagen strip (Chrono-log). The microfluidic flow device channels were oriented perpendicular to the patterned collagen strip, resulting in a 50 μm patch of collagen across the width of each channel. Whole blood was labeled with a Pacific Blue anti-human CD41 antibody (BioLegend) for 10 min, followed by the addition of 30 μg ml^−1^ Alexa Fluor 647 human plasma fibrinogen conjugate (Life Technologies) and either 1 μM NBD labeled investigational peptide, 1 μM annexin V (BioVision), or 15 USP ml^−1^ heparin. Immediately before the assay, the whole blood mixture was recalcified to 7.5 mM CaCl_2_. The whole blood was then pulled through the device channels for 10 min at wall shear rate 100 s^−1^ using a PhD Ultra syringe pump (Harvard Apparatus, Holliston, MA). Platelet aggregation, fibrin formation, and peptide accumulation were captured in real time by epifluorescence microscopy using an IX81 inverted microscope with a 40× objective (Olympus Equipment, Center Valley, PA) equipped with an Orca-R2 16-bit CCD camera (Hamamatsu, Bridgewater, NJ). Fluorescence intensities and platelet surface area coverages were measured using ImageJ (NIH, Bethesda, MD).

### Electron microscopy

Thrombi formed in the microfluidic flow assay were prepared as previously described and imaged with a JSM-7000F scanning electron microscope (JEOL, Peabody, MA) at a working distance of 6 mm and accelerating voltage of 1.5 kV^56^.

### Intravital microscopy

This procedure was performed essentially as described previously^35^. Briefly, male mice 8–12 weeks of age were anesthetized via intraperitoneal injection of ketamine/xylazine/acepromazine (100/10/2 mg kg^−1^). A cannula was introduced into the jugular vein for delivery of fluorescently labeled antibodies and peptides, and additional anesthetic as needed. The cremaster muscle was exteriorized, cleaned of connective tissue, opened and spread flat on the glass coverslip of a custom built chamber for viewing by intravital microscopy. The cremaster preparation was continuously superfused with bicarbonate buffer warmed to 36.5 °C and bubbled with 95% N_2_/5% CO_2_. The cremaster microcirculation was visualized using a BX61WI upright microscope with a 60X (0.9 NA) water immersion objective (Olympus Equipment), coupled to a CSU-X1 spinning disk confocal scanner (Yokogawa, Japan). Diode pumped solid state lasers (488 nm, 568 nm, 640 nm) with AOTF control (LaserStack, Intelligent Imaging Innovations, Denver, CO) were used as the fluorescence excitation light source. Confocal fluorescence images were acquired using an Evolve EM-CCD digital camera (Photometrics, Tucson, AZ). The microscope, confocal scanner, lasers and camera were all controlled and synchronized using SlideBook 6.0 image acquisition and analysis software (Intelligent Imaging Innovations). 30–40 μm diameter arterioles with unperturbed blood flow were selected for study. Vascular injury was induced with a pulsed nitrogen dye laser at 440 nm (NL100, Stanford Research Systems, Sunnyvale, CA) focused on the vessel wall by the microscope objective. The laser power was set to 55–65% and the laser fired at the vessel wall until a small number of red blood cells exited the lumen of the vessel (1–10 laser pulses). Anti-CD41 F(ab)_2_ fragments (0.12 μg g^−1^; clone MWReg30, BD Biosciences), and anti-fibrin antibody (0.2 μg g^−1^; clone 59D8) were infused intravenously via the jugular vein to label platelets and fibrin, respectively. Antibodies were labeled with Alexa Fluor 568 and 647 monoclonal antibody labeling kits according to the manufacturer’s instructions (Life Technologies). NBD labeled D-MARCKS ED or C2BL3-L peptide (5 mg kg^−1^) were infused at the same time as the antibodies and imaged in the 488 nm excitation channel.

### Statistics

All plots and statistical analyses were performed with Prism 6 (GraphPad, La Jolla, CA). Results are presented as means ± SD unless otherwise noted. Comparisons between two groups were performed with either two tailed Student’s *t*-tests or Mann-Whitney tests. Multiple comparisons between more than two normally distributed groups were performed with one-way analysis of variance (ANOVA) with Dunnett’s *post hoc* test. For all cases, *P* < 0.05 were considered to be significant.

### Study approval

Human whole blood (WB) was collected by venipuncture from healthy volunteers with informed consent under an Institutional Review Board-approved protocol. All experiments performed on human platelets have been described and approved by Colorado IRB (COMIRB 09-0816) and are consistent with Institutional Guidelines. The samples were de-identified after platelet preparations were made and the operator who performed the experiments worked with de-identified samples. There are no images or information in the manuscript that could lead to the identification of the donors. Animal study procedures were approved by the Institutional Animal Care and Use Committee of the University of Pennsylvania. All experiments were approved by pertinent university animal care and use committees. All methods were performed in accordance with relevant institutional guidelines and regulations.

## Electronic supplementary material

Supplementary Information

Supplementary Video S1

Supplementary Video S2
