# Supplementary material for: Peptides derived from MARCKS block coagulation complex assembly on phosphatidylserine
Source: Sci Rep. 2017 Jun 27;7:4275. doi: 10.1038/s41598-017-04494-y (PMC5487340; doi:10.1038/s41598-017-04494-y)
Supplement: Supplementary file 1 — Supplementary Information [file 41598_2017_4494_MOESM1_ESM.pdf]

Supplementary Information

Peptides derived from MARCKS block coagulation complex assembly on phosphatidylserine

Noah Kastelowitz[1], Ryo Tamura[1], Abimbola Onasoga[2], Timothy J. Stalker[3], Ormacinda R. White[1], Peter N. Brown[1], Gary L. Brodsky[2], Lawrence F. Brass[3], Brian R. Branchford[2], Jorge Di Paola[2]*, and Hang Yin[1]*

[1]Department of Chemistry & Biochemistry and the BioFrontiers Institute, University of Colorado Boulder, Boulder, Colorado, USA.

[2]Department of Pediatrics, University of Colorado School of Medicine, Aurora, Colorado, USA.

[3]Department of Medicine, University of Pennsylvania, Philadelphia, Pennsylvania, USA.

*These authors jointly supervised this work. Correspondence should be addressed to:

Hang Yin (Department of Chemistry & Biochemistry and the BioFrontiers Institute, University of Colorado Boulder, 3415 Colorado Ave., Boulder, CO 80303, USA; 303-492-6786; hubert.yin@colorado.edu).

Jorge Di Paola (Department of Pediatrics, University of Colorado School of Medicine, 12800 East 19th Ave., Aurora, CO 80045, USA; 303-724-4000; jorge.dipaola@ucdenver.edu).

Supplementary Figures

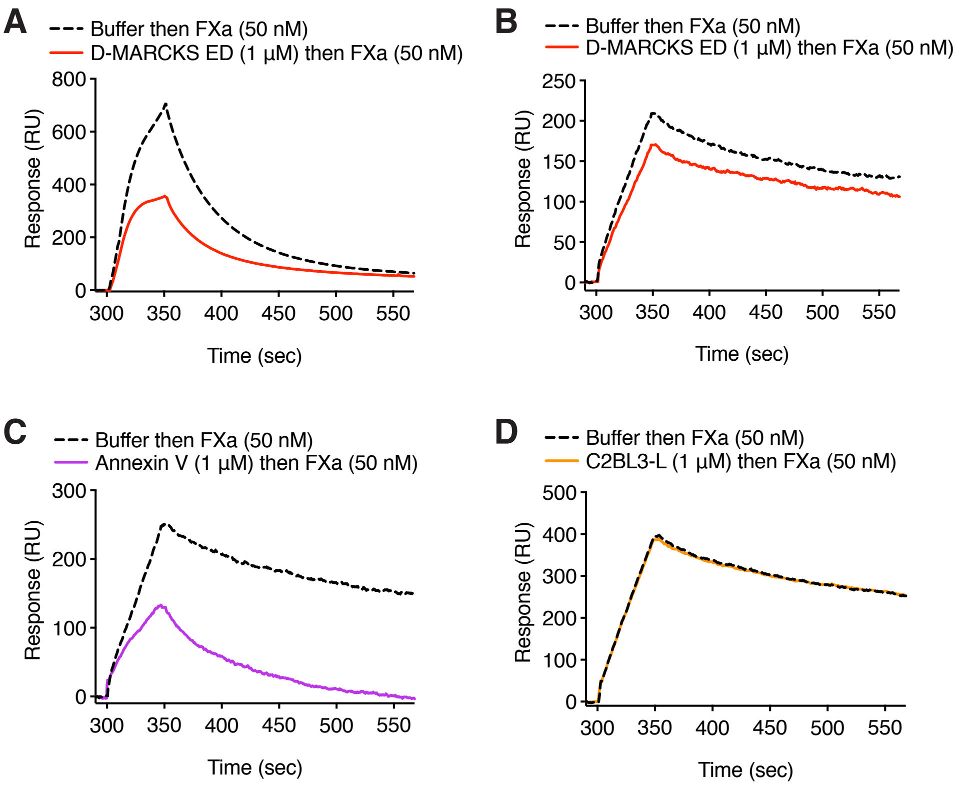

Supplementary Figure S1 SPR sensograms of FXa binding response corrected by double referencing. In each panel, the dashed black lines show an injection of 50 nM FXa at t = 300 sec that was preceded by an injection of running buffer at t = 30 sec. Following complete dissociation of FXa from the membrane surface, the solid colored lines show an injection of 50 nM FXa at t = 300 that was preceded by an injection of 1 µM D-MARCKS (**A** and **B**), annexin V (**C**), or C2BL3-L (**D**) at t = 30 sec (*n* = 3). Sensogram in (**A**) was obtained with a Biacore 3000 instrument. Sensograms in (**B**-**D**) were obtained with a BiOptix 404pi instrument.

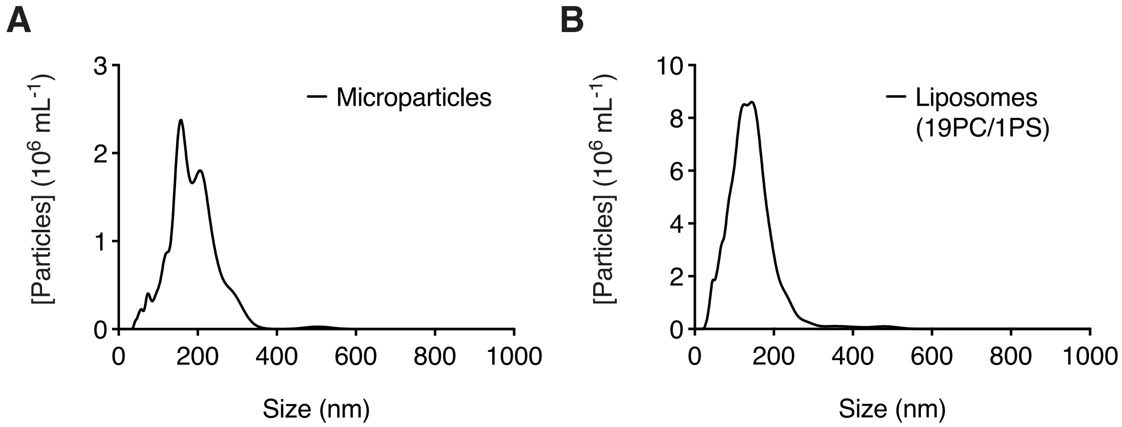

Supplementary Figure S2 Representative size distributions of prothrombinase assay biologic microparticles and synthetic liposomes characterized by nanoparticle tracking analysis. (**A**) Microparticles isolated from MDA-MB-231 human breast cancer cells. (**B**) Liposomes composed of POPC/POPS at a 19/1 ratio extruded through 100 nm pore size polycarbonate membranes.

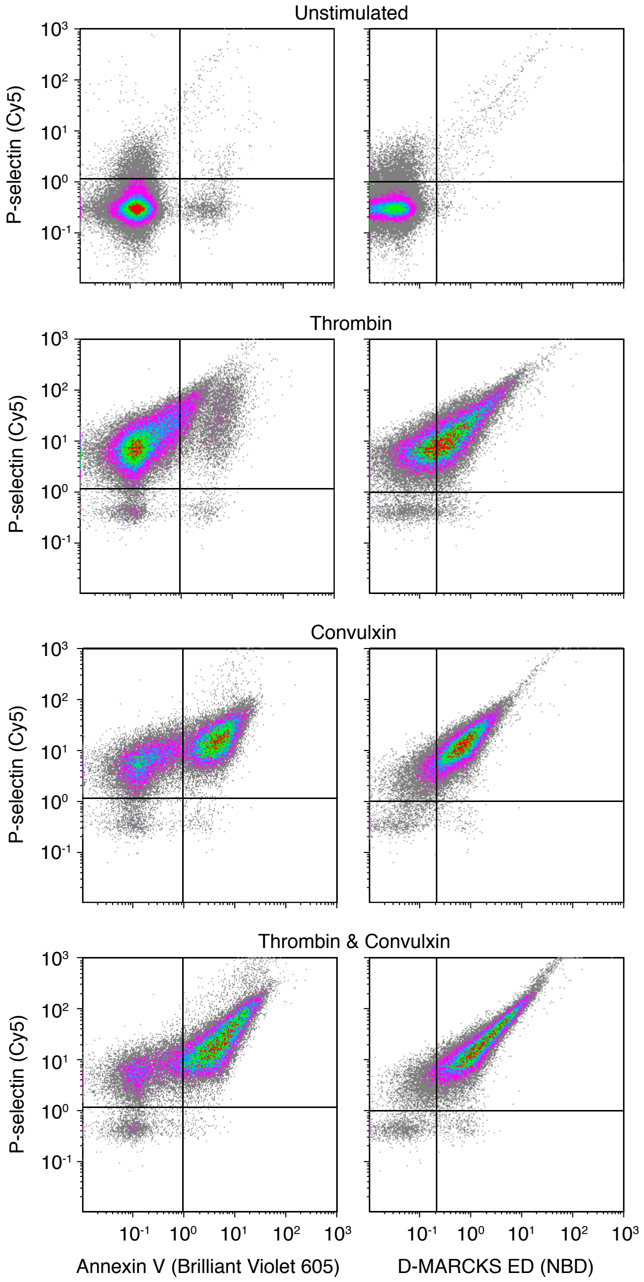

Supplementary Figure S3 Representative flow cytometry scatter plots comparing platelet annexin V (Brilliant Violet 605) or D-MARCKS ED (NBD) fluorescence intensity to P-selection (Cy5) fluorescence intensity when the platelets were left unstimulated or stimulated with thrombin, convulxin, or thrombin and convulxin (*n* = 6).

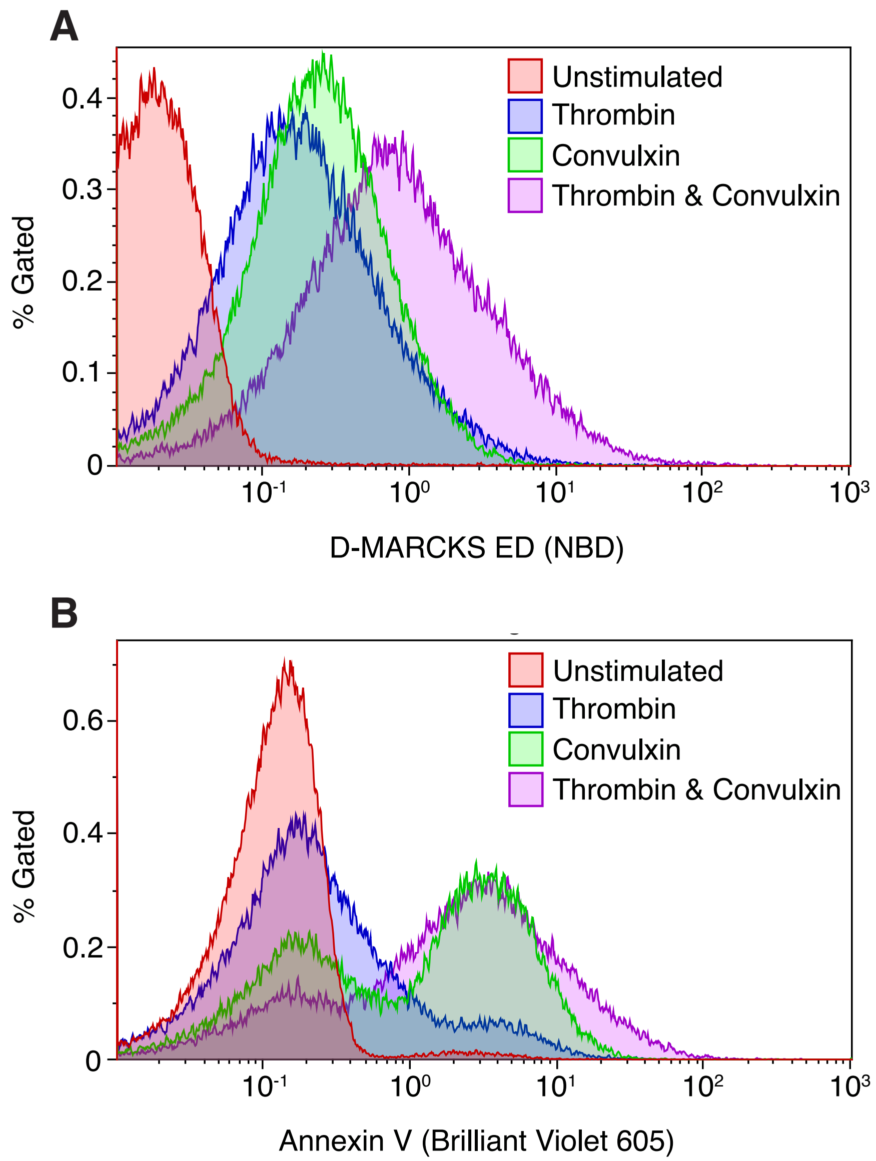

Supplementary Figure S4 Flow cytometry histograms of platelet (**A**) D-MARCKS ED (NBD) and (**B**) annexin V (Brilliant Violet 605) fluorescence intensity when platelets were left unstimulated (red) or stimulated with thrombin (blue), convulxin (green), or thrombin and convulxin (purple) (n = 6).

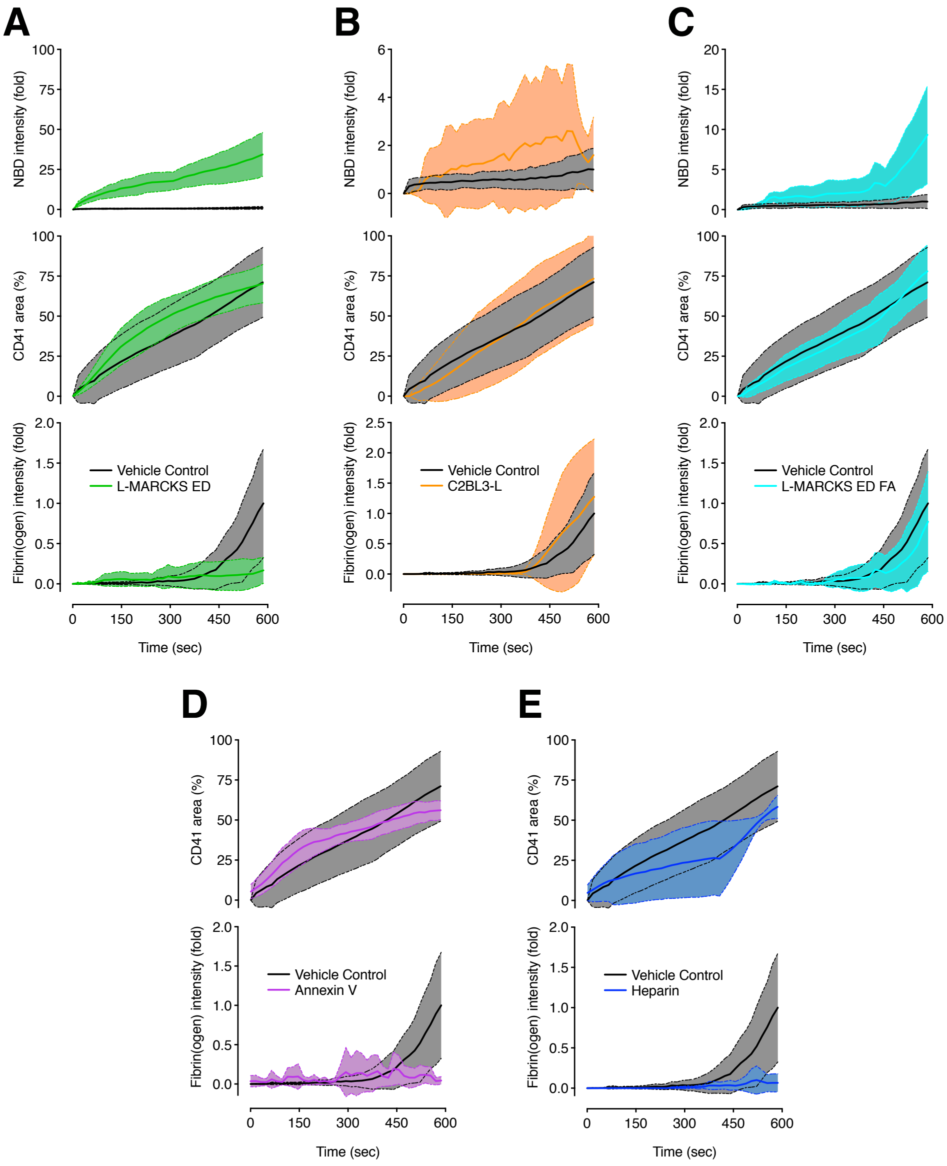

Supplementary Figure S5 Time course comparing whole blood microfluidic flow assay to peptide NBD fluorescence intensity (**A**-**C**), platelet surface area coverage, and fibrin(ogen) intensity for vehicle control to treatment with 1 μM L-MARCKS ED (**A**), C2BL3-L (**B**), L-MARCKS ED FA mutant (**C**), annexin V (**D**), or 15 USP ml^{-1} heparin (**E**) (n = 6, mean ± SD).

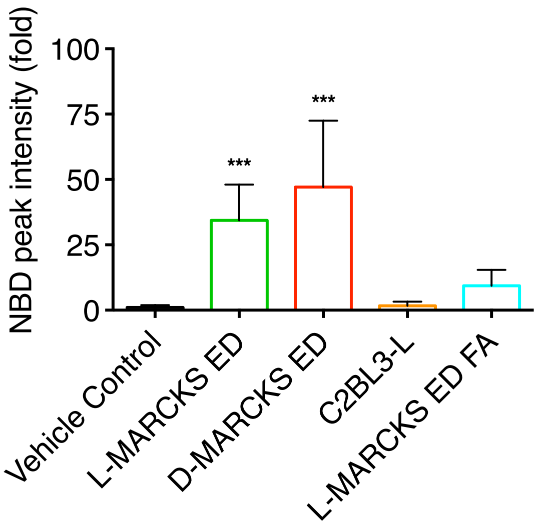

Supplementary Figure S6 Final peptide NBD fluorescence intensity values for each treatment in the microfluidic flow assay (n = 6, mean ± SD). *** $P < 0.001$ compared to vehicle control by one-way analysis of variance (ANOVA) followed by Dunnet's *post hoc* test.

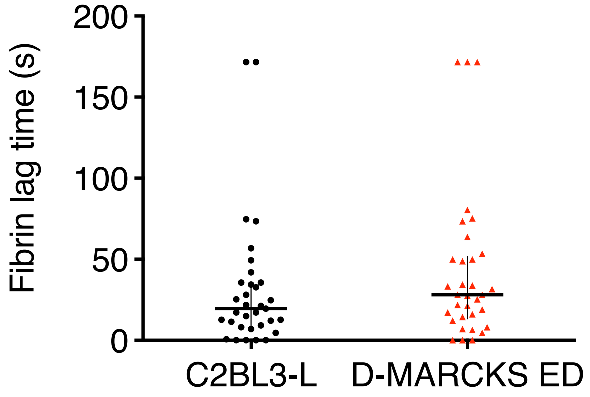

Supplementary Figure S7 Fibrin lag time, defined as time to 20 μm^2 fibrin area, in the murine intravital laser-induced microvascular injury model when treated with 5 mg kg^{-1} negative control C2BL3-L or D-MARCKS ED (*n* = 33 thrombi from 4 mice, mean ± SEM).

Supplementary Video Legends

Supplementary Video S1 Representative video of platelet accumulation and fibrin formation in the whole blood microfluidic flow assay following addition of vehicle control. Platelets are labeled with a Pacific Blue anti-human CD41 antibody and shown in blue. Fibrin(ogen) is labeled with Alexa Fluor 647 and shown in red. Video shows a 10-minute time course. Scale bar, 50 μm. (See main Figure 3).

Supplementary Video S2 Representative video of platelet accumulation, fibrin formation, and peptide labeling in the whole blood microfluidic flow assay following addition of D-MARCKS ED peptide. Platelets are labeled with a Pacific Blue anti-human CD41 antibody and shown in blue. Fibrin(ogen) is labeled with Alexa Flour 647 and shown in red. D-MARCKS ED is labeled with NBD and shown in green. Video shows a 10-minute time course. Scale bar, 50 μm. (See main Figure 3).
